# The First Case Series From Japan of Primary Headache Patients Treated by Completely Online Telemedicine

**DOI:** 10.7759/cureus.31068

**Published:** 2022-11-03

**Authors:** Masahito Katsuki

**Affiliations:** 1 Neurology, Komuginomori Headache Clinic, Shiojiri, JPN

**Keywords:** information technology, artificial intelligence, tension-type headache, online telemedicine, migraine, medication-overuse headache (moh), coronavirus disease 2019 (covid-19)

## Abstract

Background

Since March 2020, the coronavirus disease 2019 pandemic has increased the need for telemedicine to avoid in-person consultations. Online clinics for most diseases officially started in Japan in April 2022. Here, we report the cases of eight Japanese headache patients treated by completely online telemedicine for three months from the first visit.

Methodology

From the medical records between July 2022 and October 2022, we retrospectively investigated eight consecutive first-visit primary headache patients who consulted our online headache clinic via telemedicine and continued to see us via telemedicine only. The Headache Impact Test-6 (HIT-6) score, monthly headache days (MHD), and monthly acute medication intake days (AMD) were investigated over the observation period.

Results

A total of eight women were included, and the median (interquartile range) age was 30 (24-51) years. The median HIT-6 scores before, one, and three months after treatment were 63 (58-64), 54 (53-62), and 52 (49-54), respectively. MHD before, one, and three months after treatment were 15 (9-28), 12 (3-17), and 2 (2-8), respectively. AMD before, one, and three months after treatment were 10 (3-13), 3 (1-8), and 2 (0-3), respectively. Significant reductions in HIT-6 and MDH were observed three months after the initial consultation (p = 0.007 and p = 0.042, respectively). AMD was not significantly decreased at three months (p = 0.447).

Conclusions

This is the first report of Japanese patients treated by completely online telemedicine for three months from the first visit. HIT-6 and MDH can be significantly decreased at three months by only telemedicine. Online telemedicine is expected to be widely used to resolve unmet needs in headache treatment.

## Introduction

The public health issue of headaches is widespread. The two common primary headaches are migraines and tension-type headaches (TTHs), which are described in the International Classification of Headache Disorders third edition (ICHD-3) [1]. Both migraine and TTH are frequent illnesses affecting people’s quality of life, family ties, and productivity at work [2-7]. However, 59.4% of people who suffer from primary headaches never see a doctor [6]. Therefore, most headache sufferers presumably manage their pain by taking over-the-counter (OTC) medicines [7]. Additionally, when patients with headaches consult a doctor, only neuroimaging is performed to rule out organic or emergent disorders, and the diagnostic for a detailed primary headache and its treatment is insufficient, which causes patient discontent [7]. These inadequate headache medical resources and OTC medicine use may lead to medication-overuse headaches (MOHs) [3,8,9] and chronic migraine development [10]. To improve this situation, a headache awareness campaign has been performed and has raised patient awareness of consulting doctors regarding their headaches [10,11]. Therefore, patients are gradually seeking medical attention; however, there are still few headache-specialized clinics, and patients with headaches do not want to take time off from work to consult doctors during the day on weekdays [11].

Since March 2020, the coronavirus disease 2019 (COVID-19) pandemic has increased the need for telemedicine to avoid face-to-face consultations [12,13]. In this context, online clinics for most diseases officially started in Japan in April 2022. The safety and efficacy of virtual consultation for primary headaches are not different between telemedicine and face-to-face medicine [14]. There are growing expectations that telemedicine will be used to provide video-based treatment in place of traditional face-to-face treatment. However, there are few reports of headache clinics continuing to provide online headache care from the initial visit. Therefore, in this study, we describe the cases of eight Japanese headache patients treated by completely online telemedicine for three months from the first visit.

## Materials and methods

Study population

From the medical records between July 2022 and October 2022, we retrospectively investigated eight consecutive first-visit primary headache patients who consulted our online headache clinic via telemedicine and continued to see us via telemedicine only. All patients suffered from headaches at least 90 days before their first visit. The headache diagnosis was based on the ICHD-3 [1]. Episodic migraine (EM), chronic migraine (CM), TTH, and MOH were diagnosed.

We obtained electronically written informed consent for this study from all patients. This retrospective study was performed following the Declaration of Helsinki.

General management

Our telemedicine was performed based on the Guidelines for the Appropriate Implementation of Online Medical Treatment, revised in January 2022 (https://www.mhlw.go.jp/content/000889114.pdf). Before seeing the patients online, we ask them to fill out the electronic medical questionnaire. While raising the red and orange flags of secondary headache [15] in mind, we see the patients through real-time, video-based communication systems using personal computers or smartphones. The patients are asked to provide a detailed medical history as part of the “pre-examination consultation,” which should be performed before telemedicine. Although there were no applicable patients in this study period, we intended to promptly refer patients to a nearby hospital if we suspected fatal secondary headaches or other conditions that would make them inappropriate for online consultation. We prepared online telemedical practice planning sheets, which described treatment strategies, responses to sudden changes or urgent situations, communication tools, and security risks, and shared them with the patients.

After diagnosing the headache based on the ICHD-3, we treated patients by referring to the Japanese Clinical Practice Guideline for Headache 2021 [7]. Depending on the severity, we prescribed acute medications such as non-steroidal anti-inflammatory drugs, triptans, and lasmiditan, and novel 5-HT receptor agonists with high affinity and selectivity for the 5-HT_1F _serotonin receptor. In addition, we also prescribed prophylactic medications, such as lomerizine, propranolol, valproic acid, amitriptyline, muscle relaxants, and Kampo medicine [16-18] (goreisan, goshuyuto, and yokukansan). The patients were referred to a nearby headache hospital if monoclonal calcitonin gene-related peptide antibodies were indicated as prophylactic medicine.

Because we could only prescribe the medicines for seven days in the first consultation during online telemedicine, the patients were reexamined on the seventh day. The medications were continued if there were no severe side effects. If necessary, a change in prescription was considered. We could prescribe medicine via online telemedicine for up to 30 days. Online reconsultation and advice were performed as needed when appropriate. We asked patients to keep electronic headache diaries, which are supervised by The Japanese Headache Society (https://www.henzutsu.net/know-the-headache/line-healthcare). We instructed them to come to our clinic or visit a nearby hospital when they were not feeling well. We explained that after the three-month follow-up, the patients would be followed up online as well as with a face-to-face consultation by our clinic or their family doctors.

Clinical variables and outcomes

We collected patients’ characteristics, such as age, sex, comorbidities, medication, and onset age of headache. Clinical data reported by electronic headache diaries were used. Monthly headache days (MHD) and monthly acute medication intake days (AMD) were defined as the monthly values over the respective observation period of 30 days. A headache day was defined as a day with any kind of headache. The Headache Impact Test-6 (HIT-6) score [19] was also investigated over the respective observation period. The prescribed prophylactic medications were also checked. The outcomes were defined as the changes in HIT-6 score, MHD, and AMD before treatment and after one or three months.

Statistical analysis

Results were presented as the median (interquartile range). A Friedman’s test and a subsequent Wilcoxon’s test were performed to compare HIT-6 score, MHD, and AMD before treatment and after one or three months. We conducted these analyses using version 28.0.0 of SPSS software (IBM Corp., Armonk, NY, USA). A two-tailed p-value of <0.05 was considered statistically significant. Bonferroni’s correction for multiple comparisons in each test was applied, but we did not apply it throughout the study [20].

## Results

General characteristics

A total of eight women were included in this study. The median age was 30 (24-51) years. Of the eight patients, three had EM, one had TTH, one had EM+MOH, one had CM+MOH, and two had EM+TTH+MOH. The median past years from the first repetitive headache was 20 (17-23) years. No one had been prescribed prophylactic medications before consulting us. We prescribed propranolol, valproic acid, amitriptyline, and Kampo medicine according to the Japanese Clinical Practice Guideline for Headache 2021 [7]. There were no major adverse effects of the prophylactic medications. We planned to adjust the prophylactic medications, provide face-to-face consultations, and refer the patients to their local doctors in the future. Other details and characteristics of each headache type are described in Table 1.

**Table 1:** Characteristics of the patients. AMD: monthly acute medication intake days; CM: chronic migraine; EM: episodic migraine; HIT-6: Headache Impact Test-6; IQR: interquartile range; TTH: tension-type headache; MHD: monthly headache days; MOH: medication-overuse headache

Case number	Age (years)	Diagnosis	Comorbidities	Medication	Onset age	0-month HIT-6	0-month MHD	0-month AMD	Prescribed prophylactic medication	1-month HIT-6	1-month MHD	1-month AMD	3-month HIT-6	3-month MHD	3-month AMD
1	28	EM	Dysmenorrhea	Low-dose pill	10	55	1	1	Propranorol, goreisan, goshuyuto	51	1	1	49	1	1
2	50	EM	Oral cancer	-	40	58	14	8	Propranorol, goreisan, goshuyuto	54	10	4	50	8	3
3	54	EM	-	-	22	64	4	4	Propranorol, goreisan	46	1	1	45	0	0
4	25	EM+MOH	Dysmenorrhea	Low-dose pill	17	63	10	12	Valproic acid, goreisan, goshuyuto	63	4	8	57	4	4
5	28	EM+TTH＋MOH	-	-	16	64	28	12	Did not want	64	28	8	Referred to another clinic		
6	26	EM+TTH+MOH	-	-	21	64	28	28	Amitriptyline, goreisan, goshuyuto	54	14	8	Referred to another clinic		
7	32	CM+MOH	-	-	26	62	16	16	Valproic acid, goreisan, goshuyuto	61	18	2	54	6	3
8	61	TTH	-	-	18	55	28	0	Goreisan, yokukansan	53	17	0	54	14	0
Median (IQR)	30 (28-51)	-	-	-	20 (17-23)	63 (57-64)	15 (9-28)	10 (3-13)	-	54 (53-62)	12 (3-17)	3 (1-8)	52 (49-54)	5 (2-8)	2 (0-3)

One patient (number five) did not want to take any prophylactic medications but OCT analgesics, so we just explained the risk of MOH and CM development. After one month, her HIT-6 score and MHD were not changed. Therefore, the patient was referred to a nearby home doctor at the one-month follow-up visit.

One patient (number six) was satisfied with the prescribed prophylactic medications. However, she requested a prescription every three months through face-to-face consultation instead of every month online consultation, which must be adhered to in online telemedicine. Therefore, the patient was referred to a nearby home doctor at the one-month follow-up visit.

Treatment efficacy

**Figure 1:**
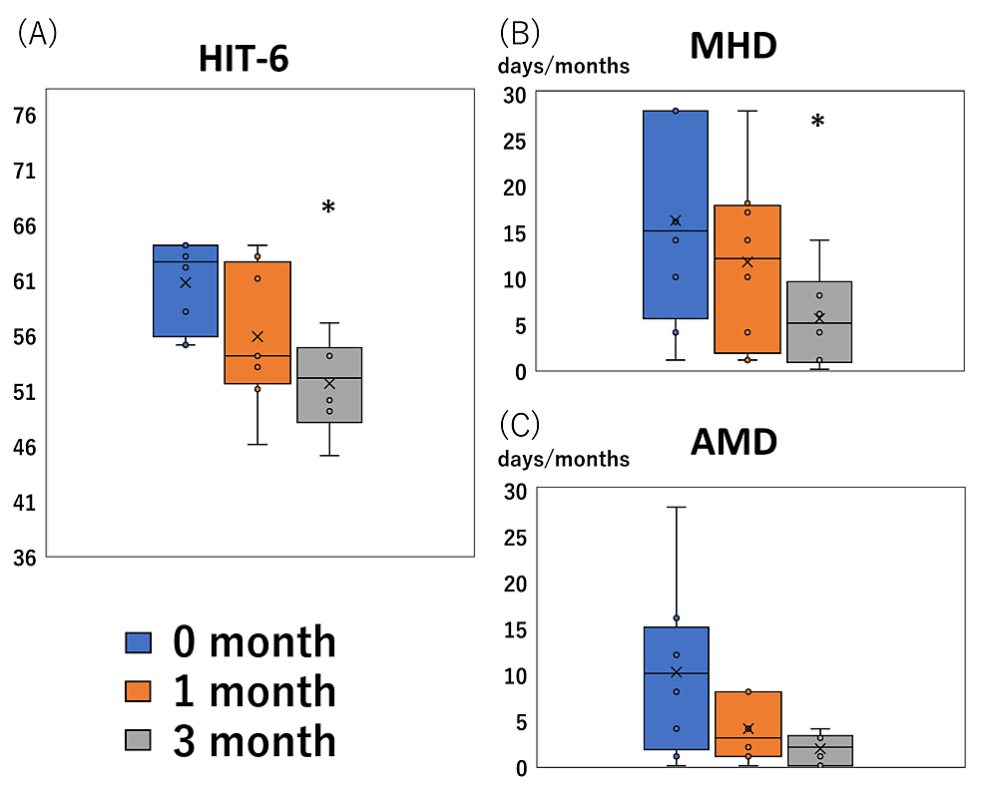
Treatment response. Boxplots of the Headache Impact Test-6 (HIT-6) score, monthly headache days (MHD), and monthly acute medication intake days (AMD) (y-axis) before treatment (0 months) and after one and three months of treatment (x-axis) are shown. HIT-6 score (A) and MHD (B) significantly improved after treatment (*; significantly decreased compared to the zero-month adjusted by Bonferroni’s correction, p < 0.05). However, AMD was not improved (C).

The median HIT-6 scores before, one, and three months after treatment were 63 (58-64), 54 (53-62), and 52 (49-54), respectively. MHD before, one, and three months after treatment were 15 (9-28), 12 (3-17), and 2 (2-8), respectively. AMD before, one, and three months after treatment were 10 (3-13), 3 (1-8), and 2 (0-3), respectively. Significant reductions in HIT-6 scores and MDH were observed three months after the initial consultation (p = 0.007 and p = 0.042, respectively) but not one month after (p = 0.250 and p = 0.937, respectively). AMD was not significantly decreased (p = 0.130 at one month and p = 0.447 at three months) (Figure 1).

## Discussion

This is the first report of Japanese patients treated by completely online telemedicine for three months from their first visit. HIT-6 score and MDH were significantly decreased at three months without a face-to-face consultation.

Benefits of online telemedicine

Telemedicine reduces the risk of infection by avoiding face-to-face encounters and can be used by a wide range of people not just patients in rural [21] and remote areas or on remote islands where in-person encounters are challenging or those who have mobility issues, such as those receiving home healthcare. Patients who are working-age and have children will have fewer appointments, but they will still be able to see doctors regularly via online telemedicine. Therefore, telemedicine allows headache specialists to provide ongoing headache care and avoids medication overuse for headache patients receiving medical treatment [7]. Clinical trials conducted over the past 10 years have shown that patients view telemedicine as valuable and cost-effective, with satisfaction levels and results comparable to those of conventional face-to-face consultations [22], with satisfaction rates and outcomes similar to traditional in-person visits [23]. Some patients may prefer to be examined via telemedicine rather than face-to-face [24] because they do not want others to know that they are visiting the hospital.

Remote monitoring is made possible by telemedicine. Implementing mobile health solutions, such as smartphone applications, has the potential to replace follow-up appointments and tracking of therapy responses completely. The use of an electronic headache diary and remote monitoring for migraine and MOH patients are superior to traditional monitoring strategies (i.e., paper diaries) in reducing headache days, the use of acute medications, and increasing adherence to treatment [25]. The results of the HIT-6 questionnaires, which are frequently utilized in clinical practice, serve as a strength in the field of remote monitoring of migraine. Using applications that have been developed and approved by science, these scores and variables can be easily shared on digital platforms for the care of patients. The applications also enable telerehabilitation and behavioral modification strategies [26]. In addition, based on the vast amount of headache recording data, so-called big data, an automated diagnostic system using artificial intelligence can further assist medical practitioners in telemedicine [13,27].

In our case, we could enjoy the benefits of telemedicine, including patients’ easy access to the doctor’s consultation despite their business, follow-up with an electronic headache diary, and the ability to obtain HIT-6 score and MMD from them as remote monitoring.

Risk of online telemedicine

The estimated number of consultations needed to miss one secondary headache with the use of telemedicine was 20,200 [14]. Additionally, among the several neurological illnesses, the only initial consultation symptoms that did not exhibit greater rates of evaluation and reinvestigation in telemedicine were headaches and suspected epileptic seizures [28]. In contrast to movement disorders, where a neurological evaluation typically informs treatment choices, medical history is critical in treating both diseases [29]. In doctor-to-patient telemedicine, however, the information obtained is dependent on the communication device and its literacy, and detailed neurological examinations are not possible. The risk of missing secondary headaches is higher than usual face-to-face consultation because neurological examination, laboratory, and radiological tests are unavailable. For these reasons, a doctor-to-patient-with-doctor method [30] is sometimes used instead of doctor-to-patient telemedicine, as in our case. This method is expected to reduce the risk of missing secondary headaches and eliminate the maldistribution of headache specialists. This style is being implemented in Japan.

There are still numerous challenges to be resolved, such as building the required infrastructure (such as solving the digital divide and installing communication networks, computer equipment, and standardized specifications), covering the costs of implementation and maintenance, providing patients and family members with adequate explanations, ensuring the security of patient video transmissions, creating a method to maintain patient medical records, and allocating qualified medical personnel. Concerns have also been raised about the inappropriate use of telemedicine, such as the sale of prescription drugs and medications without a doctor’s consultation, both of which are expressly prohibited by Japanese law [30]. Telemedicine is just getting started, and it is imperative to confirm future legislation and operational status.

Limitations of this study

First, the sample size was small, and this study was performed in a single clinic. We should be cautious about generalizing the results of our cases. Second, we did not compare the cases to the control arm. Therefore, the true differences between a completely online telemedical practice and telemedicine combined with face-to-face care were not obvious. Third, the follow-up period was short at three months, and side effects of acute and prophylactic medications can occur in the long term. Therefore, we should follow up with the patients carefully and combine face-to-face consultations and referrals to nearby physicians as appropriate, including laboratory and radiological tests. Fourth, it is also unclear when face-to-face consultation should be inserted through telemedicine-based practice. Online medical care is still in its infancy in Japan. Further studies on online telemedicine for clinical headache practice should be performed in future research.

## Conclusions

This is the first report of Japanese patients treated by completely online telemedicine for three months from their first visit. HIT-6 score and MDH were significantly decreased at three months. Expanding the use of telemedicine to other fields, paying for equipment implementation and maintenance costs, giving patients and families adequate explanations, ensuring the security of patient video transmissions, developing a method to maintain patient medical records, designating qualified medical personnel to oversee the treatment, and paying associated costs are some future challenges. Nevertheless, we think that our instances offer insightful information on how telemedicine will grow and develop in Japan.
